# Supplementary material for: Structural basis of gap-filling DNA synthesis in the nucleosome by DNA Polymerase β
Source: Nat Commun. 2025 Mar 17;16:2607. doi: 10.1038/s41467-025-57915-2 (PMC11914125; doi:10.1038/s41467-025-57915-2)
Supplement: Supplementary file 2 — Description of Supplementary Data files [file 41467_2025_57915_MOESM2_ESM.docx]

**Description of additional Supplementary Data**

**File Name:** Supplementary Data 1

**Description:** List of oligonucleotides used in this study.
